# An Enhanced Variable Two-Step Floating Catchment Area Method for Measuring Spatial Accessibility to Residential Care Facilities in Nanjing

**DOI:** 10.3390/ijerph121114490

**Published:** 2015-11-13

**Authors:** Jianhua Ni, Jinyin Wang, Yikang Rui, Tianlu Qian, Jiechen Wang

**Affiliations:** 1 Department of Geographic Information Science, Nanjing University, Nanjing 210093, China; E-Mails: neejianhua@126.com (J.N.); jywang0301@163.com (J.W.); ruiyikang@gmail.com (Y.R.); qiantianlu_nju@126.com (T.Q.); 2 Department of Resources Environment and Tourism Management, West Anhui University, Anhui Luan 237012, China; 3 Jiangsu Provincial Key Laboratory of Geographic Information Science and Technology, Nanjing 210093, China; 4 Jiangsu Center for Collaborative Innovation in Geographical Information Resource Development and Application, Nanjing 210023, China

**Keywords:** two-step floating catchment area (2FCA) method, spatial accessibility, residential care facility, catchment size

## Abstract

Civil administration departments require reliable measures of accessibility so that residential care facility shortage areas can be accurately identified. Building on previous research, this paper proposes an enhanced variable two-step floating catchment area (EV2SFCA) method that determines facility catchment sizes by dynamically summing the population around the facility until the facility-to-population ratio (FPR) is less than the FPR threshold (FPRT). To minimize the errors from the supply and demand catchments being mismatched, this paper proposes that the facility and population catchment areas must both contain the other location in calculating accessibility. A case study evaluating spatial accessibility to residential care facilities in Nanjing demonstrates that the proposed method is effective in accurately determining catchment sizes and identifying details in the variation of spatial accessibility. The proposed method can be easily applied to assess other public healthcare facilities, and can provide guidance to government departments on issues of spatial planning and identification of shortage and excess areas.

## 1. Introduction

In recent years, population aging has presented unprecedented challenges for the Chinese government, with one of the most important tasks being to make fair and equitable residential care available for elderly people [1,2]. However, providing access to elder care is a very complex challenge, influenced by many spatial factors (e.g., roads, geographic location) and non-spatial factors (e.g., social class, income and age). The Chinese government usually measures access to residential care from a non-spatial perspective, rather than a comprehensive perspective including both non-spatial and spatial factors [3]. Chinese civil administration and planning departments urgently require reliable accessibility measures to accurately identify residential care facility (RCF) shortage and excess areas.

There are many methods of accessibility measurement in the healthcare field. Potential spatial accessibility, which includes regional availability and regional accessibility, is commonly used to measure the accessibility of primary healthcare [4,5,6]. The regional availability approach is expressed as a ratio of supply to demand within a region. Government funding based on this approach is easily implemented because the region is usually predefined as an administrative boundary. However, this approach often draws sharp criticisms because it cannot reveal the detailed spatial variations within the boundary, and people within the region do not seek care beyond the region boundary [7,8,9]. The regional accessibility approach considers complex interactions between supply and demand location in different regions [10,11,12]. The gravity model is an integration of regional availability (demand-to-supply ratio) and regional accessibility (interaction between supply and demand) [13,14,15]. Although the gravity model is theoretically more sound, it involves more computation and programming and is less intuitive [9].

The two-step floating catchment area (2SFCA) method, which is essentially a special case of the gravity model, is more intuitive to interpret and more easy to calculate than the gravity model [16,17,18]. However, despite many applications in several studies, the 2SFCA method has drawn sharp criticism because it does not consider distance decay within the same given region [19,20]. The E2SFCA [15] and KD2SFCA methods [21,22] are two extended 2SFCA methods that address the distance decay problem by dividing the catchment area into several subzones, each evaluated with a different weight value according to different degrees of distance impedance [23]. However, the E2SFCA method still does not consider the distance decay within each subzone. To address this problem, the KD2SFCA method modified the E2SFCA method by applying weights based on a continuous decay function [24]. Another problem with all of the floating catchment area (FCA) frameworks is the selection of a functional form that appropriately represents spatial accessibility. To resolve this problem, Wan, *et al.* [25] proposed a three-step floating catchment area (3SFCA) method, which posited that the potential competition among the supplies and demands resulted in a “probability” that people would choose to visit any one supply site out of all the available supply sites in the catchment area. Luo [26] introduced the Huff model into the FCA method to resolve the influence of both distance impedance and supply capacity on spatial accessibility. Delamater [27] suggested that all FCA metrics were based on the assumption that supply was optimally configured to meet the needs of the demand.

The traditional FCA method is built on a fixed catchment size for all supply and demand locations. However, in actuality, there may be great differences in the capacities of different supply and demand locations, and people in urban areas often travel less distance to seek care than those in rural areas [19]. Therefore, each supply and demand location should have a different catchment size. Many studies have made efforts to address the issue of utilization by making adjustments to the fixed catchment size. Alexandrescu, *et al.* [28] defined the catchment size based on the patient flow data. Judge, *et al.* [29] used Thiessen polygons to define the catchment size. Yang, *et al.* [30] proposed varying the radius of the facility area based on the provider type or the neighborhood type to improve the 2SFCA method. McGrail and Humphreys [30] limited the catchment size of large metropolitan populations by introducing a capped access threshold. McGrail and Humphreys [20] introduced a five-level dynamic catchment size to address the problem of sudden changes in catchment size. Wang and Wheeler [31] constructed diagnosis catchment areas for all cancers based on cancer registry data and Bayesian logistic regression models.

**Figure 1:**
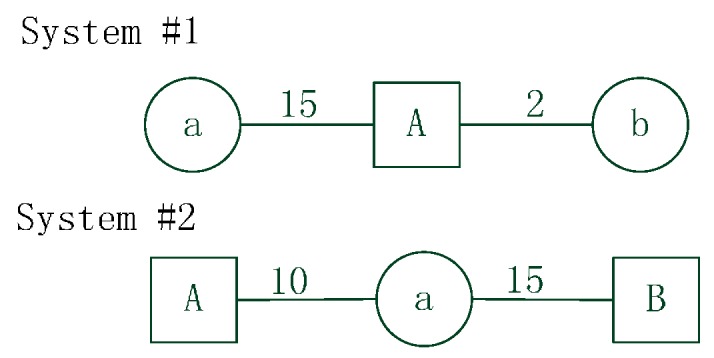
Example systems with simulated data. The systems include facility units *a*, *b* and population units *A*, *B*. The numbers between facility and population represent travel time.

Luo and Whippo [19] suggested variable two-step floating catchment area (V2SFCA) method to calculate supply and demand catchment sizes by dynamically increasing the catchment size until both demand and the supply-to-demand ratio reach their respective thresholds [32]. Although this method is more practical and effective because it utilizes a dynamic catchment size, the V2SFCA method probably calculates inaccurate supply catchment sizes because it considers single population around the supply location. However, the supply capacity also plays an important role in determination of supply catchment sizes. Inaccuracies in the determination of supply catchment sizes result in incorrect calculations of population catchment sizes that affect the final accessibility results. Furthermore, the traditional 2SFCA methods (e.g., E2SFCA, KD2SFCA, V2SFCA) do not consider intersection contradiction between supply and demand catchment areas when calculating spatial accessibility.It assumes that the population within a physician’s catchment area has adequate ability to seek care from that physician location, and that the healthcare facility within the population’s catchment area has sufficient ability to supply service to that population. However, this is not always the case. The limitation can be illustrated by two simulated system shown in Figure 1.

In System #1, suppose the catchment size of facility units *a* and *b* is 20, and the catchment size of population unit *A* is 5. According to the traditional 2SFCA methods, facility unit *a* certainly supplies service to population unit *A* because population unit *A* is within the catchment area of facility unit *a*. However, the catchment size of population unit *A* is less than the travel time between unit *a* and unit *A*. In fact, population unit *A* may rarely go to facility unit *a* for service, people probably choose facility unit *b* because facility unit *b* is closer. In System #2, suppose the catchment size of population units *A* and *B* is 20, and the catchment size of facility unit *a* is 12. According to the traditional 2SFCA methods, population unit *B* certainly goes to facility unit *a* for service because unit *a* is within the catchment area of unit *B*. However, the catchment size of facility unit *a* is less than the travel time between unit *a* and unit *B*. In fact, facility unit *a* may rarely supply service to population unit *B*.

In this paper, we propose an enhanced variable two-step floating catchment area (EV2SFCA) method, which not only overcomes the issue of inaccurate calculation of catchment size, but also minimizes the errors from the supply and demand catchments being mismatched, compared to the V2SFCA method. The second section of this paper details data sources, the procedure of the proposed method, parameters setting and spatial accessibility calculation. The third section compares and analyzes differences in the results produced by the V2SFCA and EV2SFCA methods in this case study of spatial accessibility for residential care facilities (RCFs) in Nanjing. The fourth section discusses some issues that require further study, and the last section summarizes the contributions of this paper.

## 2. Methods

### 2.1. Study Area 

The proposed method has been applied to measure the spatial accessibility of RCFs in Nanjing, China. Nanjing is the capital of Jiangsu Province, which is located slightly inland from the coast, and is a traditional large city of China. The degree of aging in Nanjing has reached 23%, with a growth rate of more than 4%. In recent years, the one-child policy and increased geographic mobility of adult children have made increasing numbers of adult children unavailable for elder care. Residential care in China today has become an alternative to familial elder care in China for those with sufficient need, money, and access, and Nanjing is experiencing unprecedented growth in the elder care service industry. According to the Nanjing Bureau of Urban Planning, which has reported on residential service layout planning in the province from 2014 to 2020, there will be 2.4 times more RCFs in 2020 than the present number, confirming that there are large gaps in RCF availability in Nanjing. For these reasons, the city of Nanjing was selected as an appropriate case study for the application of the proposed research method.

### 2.2. Study Datasets

**Figure 2:**
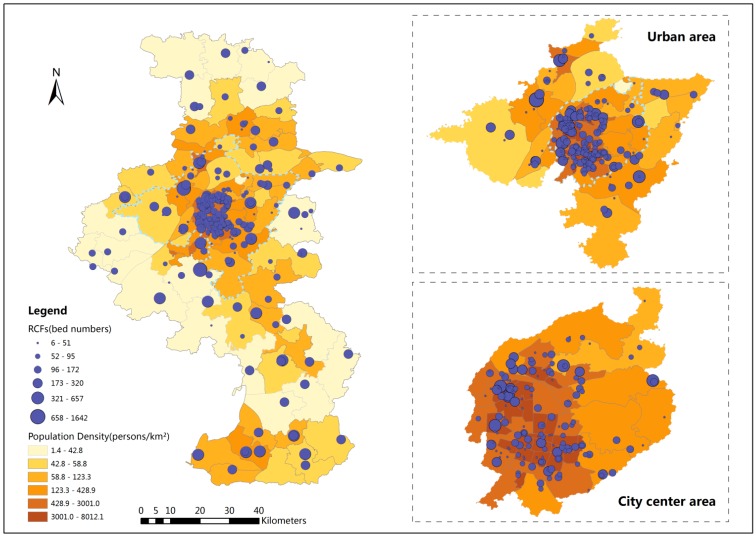
Residential care facility locations and elder population density in Nanjing.

For this research, Nanjing included both the urban and suburban areas, divided into 14 administrative districts with 118 towns/streets, as shown in Figure 2. The total population over 65 years of age is 735,036, according to the 2012 National Population Census of Nanjing. The elder population density map is shown in Figure 2. RCF data (e.g., addresses, bed numbers) and administrative data were taken from the Nanjing Municipal Civil Affair Bureau's official website [33]. According to this data, there are 275 RCFs with a total of 37,337 beds in the study area. Town and street were the smallest geographic units used for the population analysis in this study. The spatial distribution of RCFs is shown in Figure 2. The circles of different size represent different numbers of beds in the RCFs in those locations. Spatial data of the road networks are from the Nanjing Transportation Bureau. Based on the different road classes in the road network, the standard speed was set at 60 km/h for expressways, 50 km/h for main roads, 40 km/h for secondary roads, and 30 km/h for other roads.

### 2.3. Study Method

To address the limitations of the V2SFCA method, this paper presents an improved method of determining facility catchment size, as well as a method for strict intersection constraint between supply (facility) and demand (population) location*.* The new method is implemented in four steps, which are similar to theV2SFCA method:

*Step1*: The first step is to calculate the facility catchment size. For each facility location i, search all population locations within a predefined initial travel time ${\text{t}}_{0}$. Calculate the facility-to-population ratio (FPR) within the travel time ${\text{t}}_{0}$. If the ratio is less than the fixed facility-to-population ratio threshold #1 (FPRT_1_), then the travel time ${\text{t}}_{0}$ is the catchment size of that facility location i. Otherwise, the ratio exceeds the FPRT_1_, increase the travel time ${\text{t}}_{0}$ by a small increment and sum the population around this facility location within the new travel time. Calculate FPR ratio again, and repeat the process until the ratio exceeds FPRT_1_. The travel time at that point is the catchment size for that facility location i. Save the FPR_i_ for the facility location i.

*Step2*: The second step is to calculate the population catchment size. For each population location j, search all facility locations within a predefined initial travel time ${\text{t}}_{0}$ and sum FPR values of these facilities calculated in the first step. If the summed FPR is greater than the fixed facility-to-population ratio threshold #2 (FPRT_2_), the travel time ${\text{t}}_{0}$ is the catchment size of that population location j. Otherwise, increase the travel time ${\text{t}}_{0}$ by a small increment and sum the facility FPR values within the new travel time, and repeat the process until the sum is greater than FPRT_2_. The travel time at that point is considered as the population catchment size of that population location j.

*Step3*: For each facility location i, search all population locations that are within the facility catchment area (${\text{C}}_{\text{i}}$). Meanwhile, the catchment area (${\text{C}}_{\text{j}}$) of selected population location j must contain the facility location i. Then calculate the discounted FPR, ${\text{D}}_{\text{i}}$:

(1) $${\text{D}}_{\text{i}}=\frac{{\text{S}}_{\text{i}}}{\sum_{\text{j}\in \left\{{\text{d}}_{\text{ji}}\leq {\text{C}}_{\text{i}}\cap^{\text{​}}{\text{d}}_{\text{ji}}\leq {\text{C}}_{\text{j}}\right\}}{\text{P}}_{\text{j}}\times {\text{ω}}_{\text{ji}}}$$

where ${\text{S}}_{\text{i}}$ is the facility capacity of location i, ${\text{P}}_{\text{j}}$ is the population size of location j, ${\text{d}}_{\text{ji}}$ is the travel time between j and i, ${\text{ω}}_{\text{ji}}$ is the weight value for the travel time calculated from Gaussian function.

*Step 4*: For each population location j, search all facility locations that are within the population catchment area (${\text{C}}_{\text{j}}$). Meanwhile, the catchment area (${\text{C}}_{\text{i}}$) of selected facility location i must contain the population location j. Then sum the discounted FPR derived in Step 3, ${\text{A}}_{\text{j}}$:

(2) $${\text{A}}_{\text{j}}=\sum_{\text{i}\in \left\{{\text{d}}_{\text{ij}}\leq {\text{C}}_{\text{i}}\cap^{\text{​}}{\text{d}}_{\text{ij}}\leq {\text{C}}_{\text{j}}\right\}}{\text{D}}_{\text{i}}\times {\text{ω}}_{\text{ij}}$$

where ${\text{A}}_{\text{j}}$ represents the accessibility of the population location i, ${\text{D}}_{\text{i}}$ is the discounted FPR calculated in Step 3, ${\text{ω}}_{\text{ij}}$ is the weight value for the travel time calculated from Gaussian function.

There are a total of four steps in the new method. The first two steps determine the facility and population catchment sizes. The V2SFCA method calculates supply catchment sizes by incrementally (Δt) increasing the catchment until a base population (BP) threshold is met from a predefined initial travel time (${\text{t}}_{0}$). In the proposed new method, the only difference is that we replace the single population threshold with FPRT_1_ as the standard of facility catchment size. FPRT_1_ represents a ratio of facility capacity to potential serving population. If the ratio is equal to 10/1000, it means that when there are 10 beds or physicians in a facility, about 1000 people may go there for service. Population catchment size calculation in the new method is similar to the V2SFCA method. They all calculate population catchment sizes by incrementally (Δt) increasing the catchment until FPRT_2_ (or PPR in V2SFCA) is met from a predefined initial travel time (${\text{t}}_{0}$). PPR or FPRT_2_ represents desired target ratio of facility to population. If the ratio is equal to 30/1000, it means that 1000 people should own 30 beds or physicians serving them.

The last two steps are to calculate the discounted FPR and the spatial accessibility, which is similar to steps in the V2SFCA method. However, in the third step of the V2SFCA method, it only requires that the population locations should be within the facility catchment area. But the facility location may be outside the population catchment area. Similarly, in the fourth step of the V2SFCA method, the facility locations are requested to be within the population catchment area, but the population location may be outside the facility catchment area. In contrast, the new method sets strict constraints to ensure facility and population catchment areas must both contain the other location and to minimize the errors when the supply and demand catchments are mismatched.

### 2.4. Parameters Setting and Calculation

The Nanjing Municipal Civil Affair Bureau in China [34] has suggested twenty minutes as an appropriate travel time for elderly people to arrive at the nearest RCF. Therefore, this study used the ratio of average bed numbers of all RCFs to average elderly population within twenty minutes of the facility location as the ${\text{FPRT}}_{1}$ (135/50,000, average bed numbers of all RCFs is 135 and average elderly population within twenty minutes is 50,000) in Step 1. The Ministry of Civil Affairs of China [35] also suggested the standard of thirty beds per thousand elderly people, and therefore we used 30/1000 as the ${\text{FPRT}}_{2}$ in Step 2. Travel time between the population and facility location was implemented using the ESRI OD Cost Matrix function of the ArcGIS 10.1 Network Analyst model. For this study, we adopted two hours as the maximum travel time, resulting in 30,024 OD pairs.

The V2SFCA method adopts the simple stepwise Gaussian function to calculate the weights of (1.00, 0.42, 0.03) for the three zones (e.g., if a catchment is 45 min, then zone 1 is within 15 min, zone 2 from 15 to 30 min and zone 3 from 30 to 45 min) [15,19]. To reveal the much more detailed spatial distribution, here we adopted the continuous Gaussian function as the distance decay function. Spatial accessibility may be sensitive to the β value representing the distance impedance coefficient. In healthcare accessibility studies, the value of β in the Gaussian function is usually set to be greater than 1 [19,26]. However, the weight may decay slowly for measuring accessibility to RCFs because of the lower travel frequency [36,37]. For this study, we set β=1 for the Gaussian function [36,37], and used the Visual Basic developing tool to calculate the weighted travel time and spatial accessibility of each population location.

## 3. Results

**Figure 3:**
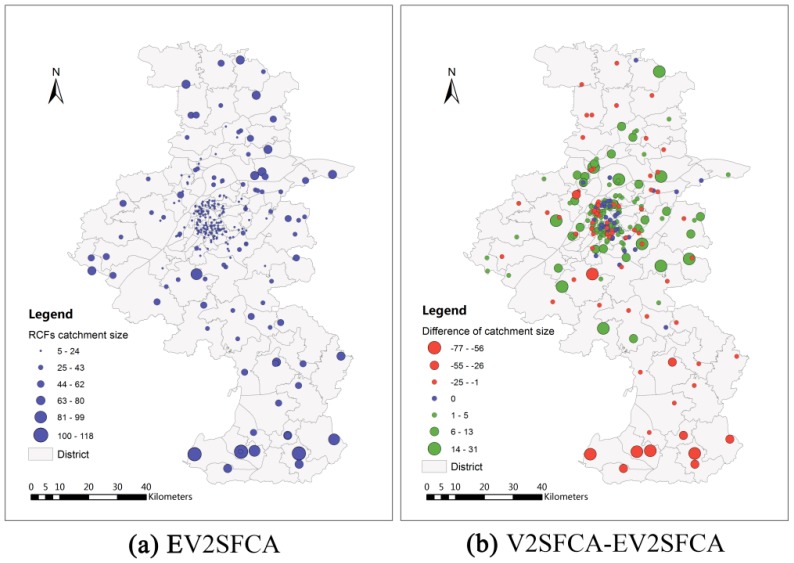
Facility catchment sizes calculated by EV2SFCA method (**a**) and facility catchment size differences between V2SFCA and EV2SFCA methods (**b**).

**Figure 4:**
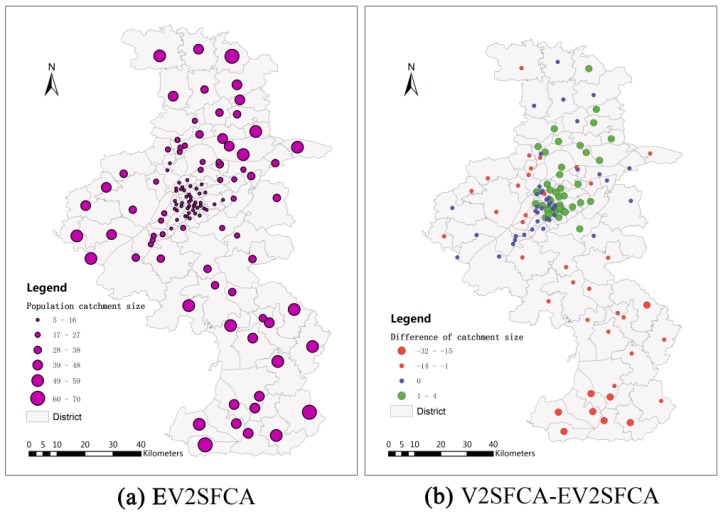
Population catchment sizes calculated by EV2SFCA (**a**) and population catchment size differences between V2SFCA and EV2SFCA methods (**b**).

Table 1 shows statistics of facility and population catchment sizes determined by the EV2SFCA method with ${\text{FPRT}}_{1}$=135/50,000, ${\text{t}}_{0}$=5 min, Δt=1 min and ${\text{FPRT}}_{2}$=30/1000 and the V2SFCA method with BP=50,000 (*i.e.*, average elderly population within twenty minutes), ${\text{t}}_{0}$=5 min, Δt=1 min and PPR=30/1000 [19]. The facility catchment sizes using V2SFCA method range from 5 min to 67 min with an average of 25.53 min and standard deviation of 13.95 min. The facility catchment sizes using EV2SFCA method have a greater range, from 5 min to 118 min with an average of 27.71 min and standard deviation of 23.88 min. The population catchment sizes using V2SFCA method range from 5 min to 63 min with an average of 23.20 min and standard deviation of 15.08 min. The facility catchment sizes using EV2SFCA method also have a greater range, from 5 min to 119 min with an average of 25.49 min and standard deviation of 17.54 min. This shows that the new method identifies more details relevant to the determination of both facility and population catchment sizes. Figure 3a and Figure 4a show the facility and population catchment sizes respectively calculated by the EV2SFCA methods. It is apparent that the catchment sizes for both facility and population locations are smaller in urban areas and larger in suburban areas. This is because the urban bed resources are more concentrated than suburban bed resources, and people in suburban areas spend more time to obtain service than that in urban areas.

Figure 3b shows the differences of facility catchmentsize between V2SFCA and EV2SFCA methods. As apparent in Figure 3b, the facility catchment sizes calculated by the V2SFCA method tend to be larger in urban areas and smaller in suburban areas. This is because V2SFCA method determines the facility catchment sizes based on single population around the facility, without regard to the capacity of the facility. But in fact, the facility catchment size is usually proportional to the number of beds. Residential care facilities with more beds may have larger catchment sizes. Therefore, the proposed method uses the ratio of facility capacity to population instead of the single population around the facility. In urban areas, the population is so dense that the actual facility catchment sizes are smaller than those calculated by the V2SFCA method. On the other hand, the actual facility catchment sizes in sparsely populated suburban areas are larger than that calculated using the V2SFCA method. Figure 3b demonstrates that the V2SFCA method tends to overestimate the facility catchment sizes in urban areas and underestimate those in suburban areas. Figure 4b shows the differences of population catchmentsize between V2SFCA and EV2SFCA methods. As shown in Figure 4b, the population catchment sizes calculated by the V2SFCA method tend to be larger in urban areas and smaller in suburban areas. This is the case because the catchment sizes of facility tend to underestimatein suburban areas, which leads to underestimate to facility service capacity and larger ratio of facility-to-population in the first step of V2SFCA method. Then, the sum of PPR reaches the specified ratio threshold in the second step using less travel time in suburban areas. In contrast, the catchment sizes of facility tend to overestimate in urban areas, which leads to overestimate to facility service capacity and smaller ratio of facility-to-population in the first step of V2SFCA method. Then, the sum of PPR reaches the specified ratio threshold in the second step using more travel time in urban areas.

**Figure 5:**
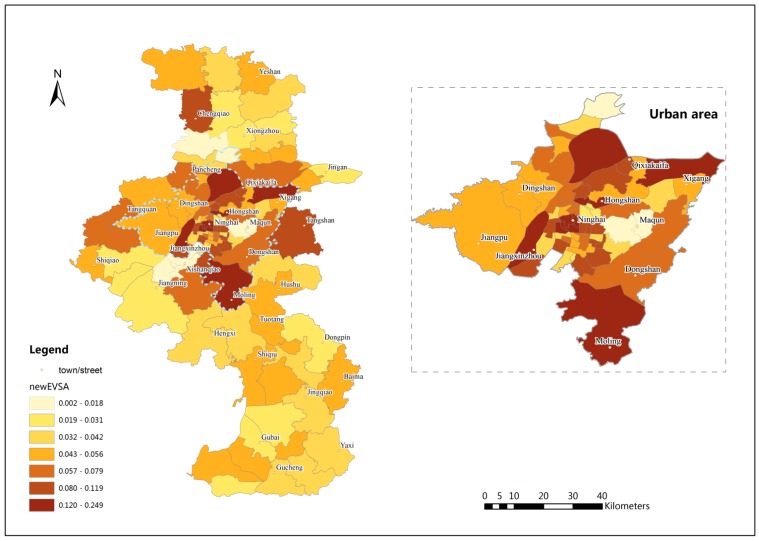
Spatial distribution of accessibility results using EV2SFCA method.

**Figure 6:**
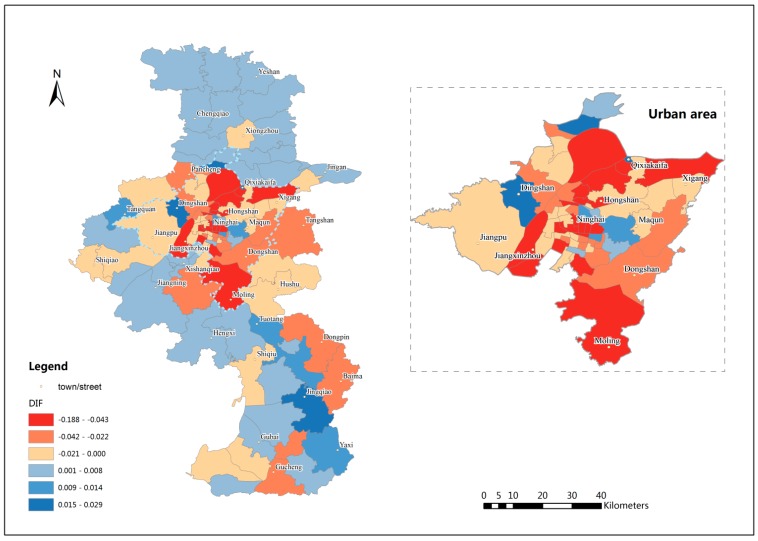
Spatial accessibility differences between EV2SFCA and V2SFCA methods.

Figure 5 shows the spatial accessibility results in the study area using EV2SFCA method and Figure 6 shows spatial accessibility differences between EV2SFCA and V2SFCA methods. The EV2SFCA method generates some different spatial accessibility patterns relative to the V2SFCA method. From Figure 6, it is clear that the accessibility scores using the EV2SFCA method tend to be higher in and around city center areas than those using the V2SFCA method. This is because the V2SFCA method overestimates facility catchment sizes in urban areas, causing exaggeration of the service capability supplied by urban RCFs to suburban population. Then the number of beds available to the urban population is underestimated in the V2SFCA method. Therefore, the accessibility scores tend be higher than those predicted by the V2SFCA method in urban areas. 

**Table 1:** Statistics of facility and population catchment sizes determined by V2SFCA and EV2SFCA methods.

Object Type	Method	Min.	Max.	Avg.	Std. Dev.
**Facility**	V2SFCA	5	67	25.53	13.95
	EV2SFCA	5	118	27.71	23.88
**Population**	V2SFCA	5	63	23.20	15.08
	EV2SFCA	5	70	25.49	17.54

In addition, the V2SFCA method does not account for intersection contradiction between the facility and population catchment areas, which may cause an overestimation of the facility’s capacity or the population’s ability to seek services. That is why the accessibility scores tend to be higher in suburban areas using V2SFCA method (see Figure 6). Therefore, the proposed method sets strict intersection constraint: facility and population catchment areas must both contain the other location. Although the population catchment sizes in urban areas are larger using EV2SFCA method, many intersections between suburban population and urban facilities have been filtered by the constraint. If we remove the constraint, the accessibility scores tend to be larger especially in the suburban areas. Without the proposed constraint, the suburban population is predicted to obtain more services from urban areas than they actually do, as is predicted using the V2SFCA method, even though the number of beds is too limited to serve the dense urban population. Using the EV2SFCA method, however, elderly people do not seek care from farther RCFs if the nearby service is sufficient, and an RCF does not supply service to farther populations if the number of beds at the RCF is insufficient. This is consistent with the fact that elderly people are unwilling to seek care in urban areas because of their poor mobility, as well as the favorable environment in suburban areas. Chinese people have a very strong desire expressed by the proverb: “Fallen leaves return to the roots.” This is especially so for elderly people because it is difficult for them adapt to new environments, which may even provide inadequate service resources. The strict intersection constraint of the proposed method are more logical and reasonable.

The results of the EV2SFCA method show higher accessibility scores that are predominantly concentrated in city center areas, such as Hongshan and Ninghai, which have the most developed traffic systems and relatively more beds *per capita*. However, the V2SFCA method shows indifferent accessibility scores in those areas that may be even lower than in some remote suburban areas, which is contrary to expectation. In general, the results of the proposed method show that the spatial distribution of RCFs in Nanjing is extremely uneven, especially in the urban areas. The city center areas, in the eastern section of the urban area, have the highest accessibility scores. Many towns and streets in these city center areas have achieved the goal set by the government. In contrast, the accessibility scores are very low in some eastern areas, like Maqun, and southwestern areas, like Xishanqiao. These areas are newly developing areas of Nanjing, where the infrastructure is inadequate to accommodate the higher occupancy rate. The variation in spatial accessibility fluctuates less in southern and northern suburban areas relative to urban areas.

## 4. Discussion

Although the EV2SFCA method overcomes two limitations of the V2SFCA method, several problems remain for further study. A first concern is how to select the appropriate FPR thresholds to determine population and facility catchment size. In this study, ${\text{FPRT}}_{1}$ is set by the ratio of average number of beds to average elderly population around the RCF location within the travel time recommended by NMCAB. ${\text{FPRT}}_{2}$ is derived from the standard set by MCAC. In fact, the values of ${\text{FPRT}}_{1}$ and ${\text{FPRT}}_{2}$ play an important role in the accessibility scores. A small ${\text{FPRT}}_{1}$ value tends to result in a large facility catchment size and low accessibility scores. To test the sensitivity of different ${\text{FPRT}}_{1}$ on the accessibility results, we used 135/40,000 and 135/60,000 as two different thresholds while keeping ${\text{FPRT}}_{2}$ at 30/1000 in Step2. The spatial accessibility patterns of the two results are similar to those shown in Figure 5. The large threshold tends to result in higher accessibility scores and the small threshold tends to result in lower accessibility scores, compared to the results shown in Figure 5. Similarly, to test the sensitivity of different ${\text{FPRT}}_{2}$ on the final accessibility results, we used 20/1000 and 40/1000 as two different thresholds while keeping ${\text{FPRT}}_{1}$ at 135/50,000 in Step 1. The spatial accessibility patterns using the two values are again consistent with the results shown in Figure 5, the small threshold tends to result in lower accessibility scores and the large threshold tends to result in larger accessibility scores, compared to the results shown in Figure 5. Consequently, ${\text{FPRT}}_{1}$ and ${\text{FPRT}}_{2}$ should be set according to actual circumstances. For example, in some remote rural areas in China, adult children still follow the filial piety and provide physical care for their elderly parents at home. Thresholds in these areas should be set based on actual surveys and utilization of facility.

A second concern is how to select the proper form of the decay function. Most decay functions in present research develop from the gravity model in one of three ways: as a continuous function, like the Gaussian function or gravity model; as a discrete time zone decay function, like the EV2SFCA and V2SFCA; or as a hybrid between the continuous and discretized decay models. We adopted the Gaussian function as the distance decay function to reveal the much more detailed spatial distribution in this study. Accordingly, the distance decay function form can be varied based on the accessibility type [19]. In the healthcare field, the discrete time zone decay function can be used because of less acute sensitivity to distance decay.

A third concern is that the spatial accessibility of RCFs is also influenced by the income of the elderly patients, fees of the RCFs, numbers of physicians in the RCFs, and other factors [17]. Using the Huff extended model, more comprehensive parameters can be incorporated into the EV2SFCA method to make it more applicable [38]. Future research should include additional surveys to properly address these issues.

## 5. Conclusions

Although the V2SFCA method addresses the limitations of the 2SFCA method that uses a fixed catchment size for all supply and demand locations, the issues of accurate calculation of catchment size and overestimated intersection between facility and population catchment areas have not been satisfactorily resolved. This paper presents an enhancement of the V2SFCA method, with two primary major contributions: (1) it improves the method of determining the facility catchment size to minimize the possibility of under- or overestimation of that parameter, which in turn ensures accurate calculation of the population catchment size and accessibility results; and (2) it proposes that the facility and population catchment areas must both contain the other location in calculating accessibility to minimize the errors from the supply and demand catchments being mismatched. When applying this method to evaluate spatial accessibility to residential care facilities in Nanjing, we find that it reveals more details in the variation of spatial accessibility. In general, the higher accessibility scores are mostly concentrated in the urban areas and lower accessibility scores are concentrated in suburban areas. In addition, urban areas show more variation in spatial accessibility, with some urban areas containing excessive bed resources, as in Jiangxinzhou, Ninghai, and others. These results suggest that some facilities should be relocated because the RCF resources are too concentrated. However, accessibility scores in some of the urban areas are extremely low, as in the west and southeast areas like Maqun and Xishanqiao. In these areas, the government of Nanjing city should invest more money to build more facilities to cope with the greater demand for RCF resources. In general, less variation exists in the spatial accessibility in southern and northern suburban areas. The catchment size and accessibility scores using the proposed method are more in agreement with expected values. The proposed method can be easily applied to assess other public service facilities, and can therefore provide guidance to government departments on issues of spatial planning and identification of shortage and excess areas.

## References

[1] Heying Jenny Z., Xiaotian F., Baozhen L. (2008). Placing elderly parents in institutions in urban China: A reinterpretation of filial piety. Res. Aging.

[2] Cheng Y., Rosenberg M.W., Wang W., Yang L., Li H. (2011). Aging, health and place in residential care facilities in Beijing, China. Soc. Sci. Med..

[3] Cheng Y., Rosenberg M.W., Wang W., Yang L., Li H. (2012). Access to residential care in Beijing, China: Making the decision to relocate to a residential care facility. Ageing Soc..

[4] Joseph A.E., Phillips D.R. (1984). Accessibility and Utilization: Geographical Perspectives on Health Care Delivery.

[5] Khan A.A. (1992). An integrated approach to measuring potential spatial access to health care services. Socioecon. Plann. Sci..

[6] Xi Y., Ren F., Liang S., Zhang J., Lin D.-N. (2014). Spatial Analysis of the Distribution, Risk Factors and Access to Medical Resources of Patients with Hepatitis B in Shenzhen, China. Int. J. Environ. Res. Public Health.

[7] Kleinman J.C., Makuc D. (1983). Travel for Ambulatory Medical Care. Med. Care.

[8] Wing P., Reynolds C. (1988). The availability of physician services: A geographic analysis. Health Serv. Res..

[9] Luo W., Wang F. (2003). Measures of Spatial Accessibility to Health Care in a GIS Environment: Synthesis and a Case Study in the Chicago Region. Environ. Plann. B.

[10] Huff D.L. (1963). A Probabilistic Analysis of Shopping Center Trade Areas. Land Econ..

[11] Huff D.L. (1964). Defining and Estimating a Trading Area. J. Marketing.

[12] Meade M.S., Earickson R. (2000). Medical Geography.

[13] Joseph A.E., Bantock P.R. (1982). Measuring potential physical accessibility to general practitioners in rural areas: A method and case study. Soc. Sci. Med..

[14] Shen Q. (1998). Location characteristics of inner-city neighborhoods and employment accessibility of low-wage workers. Environ. Plann. B.

[15] Luo W., Qi Y. (2009). An enhanced two-step floating catchment area (E2SFCA) method for measuring spatial accessibility to primary care physicians. Health Place.

[16] Radke J., Mu L. (2000). Spatial Decompositions, Modeling and Mapping Service Regions to Predict Access to Social Programs. Geogr. Inform. Sci..

[17] Wang F., Luo W. (2005). Assessing spatial and nonspatial factors for healthcare access: Towards an integrated approach to defining health professional shortage areas. Health Place.

[18] Cervigni F., Suzuki Y., Ishii T., Hata A. (2008). Spatial Accessibility to Pediatric Services. J. Commun. Health.

[19] Luo W., Whippo T. (2012). Variable catchment sizes for the two-step floating catchment area (2SFCA) method. Health Place.

[20] McGrail M.R., Humphreys J.S. (2014). Measuring spatial accessibility to primary health care services: Utilising dynamic catchment sizes. Appl. Geogr..

[21] Guagliardo M. (2004). Spatial accessibility of primary care: Concepts, methods and challenges. Int. J. Health Geogr..

[22] Dai D., Wang F. (2011). Geographic disparities in accessibility to food stores in southwest Mississippi. Environ. Plann. B.

[23] Cutumisu N., Spence J.C. (2012). Sport Fields as Potential Catalysts for Physical Activity in the Neighbourhood. Int. J. Environ. Res. Public Health.

[24] Langford M., Fry R., Higgs G. (2011). Measuring transit system accessibility using a modified two-step floating catchment technique. Int. J. Geogr. Inf. Sci..

[25] Wan N., Zou B., Sternberg T. (2012). A three-step floating catchment area method for analyzing spatial access to health services. Int. J. Geogr. Inf. Sci..

[26] Luo J. (2014). Integrating the Huff Model and Floating Catchment Area Methods to Analyze Spatial Access to Healthcare Services. T. GIS.

[27] Delamater P.L. (2013). Spatial accessibility in suboptimally configured health care systems: A modified two-step floating catchment area (M2SFCA) metric. Health Place.

[28] Alexandrescu R., O’Brien S., Lyons R., Lecky F., the Trauma Audit and Research Network (2008). A proposed approach in defining population-based rates of major injury from a trauma registry dataset: Delineation of hospital catchment areas (I). BMC Health Serv. Res..

[29] Judge A., Welton N.J., Sandhu J., Ben-Shlomo Y. (2009). Geographical variation in the provision of elective primary hip and knee replacement: the role of socio-demographic, hospital and distance variables. J. Public Health.

[30] Yang D.-H., Goerge R., Mullner R. (2006). Comparing GIS-Based Methods of Measuring Spatial Accessibility to Health Services. J. Med. Syst..

[31] Wang A., Wheeler D.C. (2015). Catchment Area Analysis Using Bayesian Regression Modeling. Cancer Inform..

[32] McGrail M.R. (2012). Spatial accessibility of primary health care utilising the two step floating catchment area method: An assessment of recent improvements. Int. J. Health Geogr..

[33] Nanjing Municipal Civil Affair Bureau Summary of Nanjing Residential Care Institutions in 2012. http://www.njmz.gov.cn/mzj/33719/ylfw/bszn_63466.

[34] Ministry of Civil Affairs of China China’s first Community and Pension Forum Held in Nanjing. http://www.mca.gov.cn/article/zwgk/gzdt/201503/20150300790891.shtml.

[35] Ministry of Civil Affairs of China Notification on Accelerating Health and Residential Care Services Construction. http://www.mca.gov.cn/article/zwgk/fvfg/shflhshsw/201409/20140900701503.shtml.

[36] Tao Z., Cheng Y., Dai T. (2014). Measuring spatial accessibility to residential care facilities in Beijing. Prog. Geogr..

[37] Tao Z., Cheng Y., Dai T., Rosenberg M.W. (2014). Spatial optimization of residential care facility locations in Beijing, China: Maximum equity in accessibility. Int. J. Health Geogr..

[38] Huff D., McCallum B.M. (2008). Calibrating the Huff Model Using ArcGIS Business Analyst.

